# Association between systemic immune-inflammation index and postoperative optical quality in the early recovery phase after cataract surgery: a retrospective study

**DOI:** 10.3389/fmed.2026.1837927

**Published:** 2026-06-12

**Authors:** Huaixiao Zang, Kaiqiang Xu, Dong Shi

**Affiliations:** 1 Department of Ophthalmology, The Fourth Affiliated Hospital of China Medical University, Shenyang, Liaoning, China; 2 Qinhuangdao Center for Disease Control and Prevention, Qinhuangdao, Hebei, China

**Keywords:** cataract surgery, modulation transfer function, objective scatter index, optical quality, systemic immune-inflammation index

## Abstract

**Objective:**

Early postoperative optical quality varies among patients after cataract surgery. Systemic inflammation is increasingly recognized as a factor influencing postoperative recovery. This study aimed to investigate the association between preoperative systemic immune-inflammation index (SII), defined as neutrophil count × platelet count / lymphocyte count, and early objective optical quality following phacoemulsification cataract surgery.

**Methods:**

This retrospective study included 110 eyes of 110 patients who underwent uneventful phacoemulsification with monofocal intraocular lens implantation. The primary outcome for this analysis was internal modulation transfer function (MTF) average height, total-eye MTF average height and corneal MTF average height were treated as secondary outcomes. Linear mixed-effects models with mean-centered log2-SII were used to analyze the relationship between SII and MTF average height. Exploratory analyses included Strehl ratio (SR), higher-order aberrations (HOAs), dysfunctional lens index (DLI) and objective scatter index (OSI). Intergroup differences were compared at each postoperative time point.

**Results:**

Higher preoperative log2-SII was significantly associated with lower internal MTF average height at postoperative 1 week (β = −0.042, 95%CI: -0.069 to -0.015, *P* = 0.002) and 1 month (β = −0.039, 95%C: -0.066 to -0.013, *P* = 0.004), but not at 1 day (*P* = 0.299). The log2-SII* time interaction was significant at 1 week (*P* = 0.032) and borderline at 1 month (*P* = 0.043). No consistent associations were found for total-eye or corneal MTF. In exploratory analyses (median-split), the low-SII group showed better internal Strehl ratio at 1 week and 1 month, lower total higher-order aberrations and secondary astigmatism at 1 month, as well as higher dysfunctional lens index and lower objective scatter index at 1 month (all *P* < 0.05). Postoperative visual acuity did not differ between groups at any time point (*P* > 0.05).

**Conclusion:**

Higher preoperative SII was associated with less favorable early postoperative internal MTF average height. Exploratory analyses suggested potential differences in SR, HOAs, DLI, and OSI, but these findings require confirmation in future studies. Despite comparable visual acuity, systemic inflammatory status may contribute to interindividual variability in early optical recovery after cataract surgery.

## 1 Introduction

Cataract remains a leading cause of visual impairment in older adults, and phacoemulsification with intraocular lens (IOL) implantation is widely performed to restore vision (1). With advances in surgical techniques and IOL design, patient expectations have increasingly extended beyond visual acuity toward overall visual quality (2). Many patients achieve satisfactory postoperative best-corrected visual acuity while still reporting differences in visual clarity, contrast, or overall visual comfort (3, 4). This discrepancy has increased interest in objective optical quality as a more comprehensive indicator of postoperative visual recovery.

Early postoperative optical quality is shaped by multiple factors. Local ocular conditions, such as tear film stability, corneal status, IOL-related factors, retinal function, and surgical variables, all contribute to postoperative visual performance (5–8). Yet these local determinants may not fully explain why recovery speed varies among patients after apparently uncomplicated surgery. It is therefore reasonable to consider whether systemic background status also influences the early recovery process (9). Routine blood testing is widely available before cataract surgery, but single hematologic indices often provide limited information on subclinical inflammatory status (10). The systemic immune-inflammation index (SII), derived from platelet, neutrophil, and lymphocyte counts, has been proposed as an integrated marker reflecting the balance between inflammatory activity and immune regulation (11). Recent studies have linked elevated SII to several ophthalmic conditions, including endophthalmitis, pseudoexfoliation syndrome, retinal vein occlusion, dry eye disease, and cataract (12–16). Even so, whether this systemic marker is related to postoperative optical recovery after cataract surgery remains uncertain.

Most previous studies on systemic inflammatory markers in cataract surgery have focused on postoperative inflammation-related complications or on conventional visual outcomes. Evidence regarding objective optical quality, especially during the early postoperative period, remains limited. This early phase is clinically important because optical quality continues to change before reaching relative stability, and these changes may not be captured by visual acuity testing alone. In this retrospective study, postoperative optical quality was evaluated using objective parameters measured with iTrace and OQAS II. The assessed metrics include MTF, SR, HOAs, DLI, and OSI, at postoperative 1 day, 1 week and 1 month. The aim of this study was to determine whether preoperative SII was associated with early objective optical quality after cataract surgery.

## 2 Materials and methods

### 2.1 Study population

**FIGURE 1:**
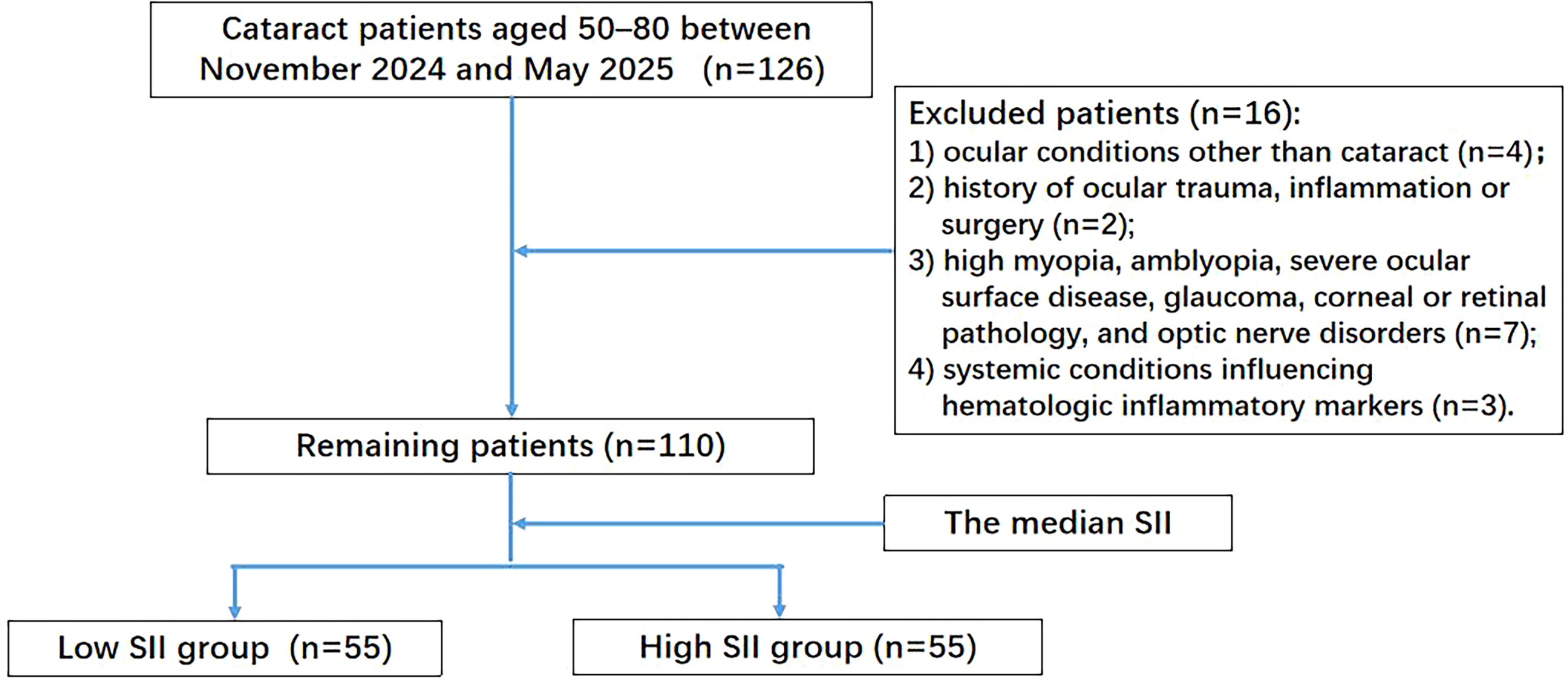
Research flowchart of the study.

This retrospective study included 126 eyes from 126 patients who underwent cataract surgery with IOL implantation at the Department of Ophthalmology, the Fourth Affiliated Hospital of China Medical University (Shenyang, China) between November 2024 and May 2025. The inclusion criteria were: (1) age between 50 and 80 years; (2) diagnosis of age-related cataract scheduled for phacoemulsification; (3) axial length of 21.0–26.0 mm, corneal astigmatism less than 1.5 diopters, endothelial cell density greater than 2,000 cells per square millimeter; (4) normal pupillary function. The exclusion criteria were: (1) ocular conditions other than cataract; (2) history of ocular trauma, inflammation, or surgery (including refractive or intraocular procedures); (3) high myopia, amblyopia, severe ocular surface disease, glaucoma, corneal or retinal pathology, and optic nerve disorders; (4) systemic conditions influencing hematologic inflammatory markers (e.g., autoimmune disease, hematologic disorders). In strict accordance with the exclusion criteria, we excluded with ocular conditions other than cataract (*n* = 4), history of ocular trauma, inflammation orsurgery (*n* = 2), high myopia, amblyopia, severe ocularsurface disease, glaucoma, corneal or retinalpathology, optic nerve disorders (*n* = 7), and systemic conditions influencinghematologic inflammatory markers (*n* = 3). In addition, to ensure the reliability of the results, we removed all unclear or missing data. A total of 16 patients were excluded. Based on strict application of the inclusion and exclusion criteria, a total of 110 eyes from 110 patients were finally included. This study was reviewed and approved by the Ethics Committee of the Fourth Affiliated Hospital of China Medical University (EC-2025-KS-185) and all participants provided written consent. The study selection process was illustrated in Figure 1.

### 2.2 Data collection

Clinical data were retrospectively collected from medical records. The aim of this study was to determine whether preoperative SII was associated with early objective optical quality after cataract surgery.

Complete Blood Count: A 3 mL fasting venous blood sample was collected from each patient within one day before surgery. Complete blood count parameters were measured using an automated hematology analyzer (Sysmex XN-9000, Japan).

Early objective optical quality: (1) Preoperative ophthalmic evaluation: Examinations included best-corrected visual acuity (BCVA), slit-lamp biomicroscopy, intraocular pressure (IOP) measurement, and corneal topography (Pentacam, Oculus, Wetzlar, Germany). Biometric parameters, including axial length (AL), anterior chamber depth (ACD), and corneal curvature, were measured using the IOLMaster (Carl Zeiss Meditec, Jena, Germany). Fundus status was assessed using scanning laser ophthalmoscopy (SLO) and optical coherence tomography (OCT). 2) Postoperative assessment: Postoperative data were recorded at 1 day, 1 week, and 1 month after surgery. At each visit, BCVA was recorded at 5 m using a logarithm of the minimum angle of resolution (logMAR) chart. Objective optical quality parameters were obtained using the iTrace aberrometer (Tracey Technologies, Houston, TX, United States) and the Optical Quality Analysis System II (OQAS II; Visiometrics SL, Terrassa, Spain). The iTrace provided HOAs (including total HOA, coma, spherical aberration, secondary astigmatism, and trefoil), MTF, DLI, and point spread function (PSF) (17). MTF values were obtained at spatial frequencies of 5, 10, 15, 20, 25, and 30 cycles per degree (c/d), and PSF was expressed as the Strehl ratio (SR), with 1.0 representing an ideal optical system. Both MTF and SR measurements were further decomposed by the iTrace system into three optical components (total eye, internal, and corneal), which facilitated the evaluation of anterior versus intraocular contributions to optical quality. The OQAS II was used to measure OSI. During testing, refractive errors were fully corrected: spherical error was automatically compensated by the device, while cylindrical error was partially corrected using external trial lenses (18). All optical quality measurements were analyzed at a pupil diameter of 3.0 mm. Examinations were performed in the same examination room by the same group of experienced technicians using identical instrument settings to ensure measurement consistency.

### 2.3 Surgical procedure and perioperative medication

All patients were admitted and managed according to the same perioperative protocol. Topical gatifloxacin was administered four times daily starting 1 day before surgery. On the day of surgery, mydriasis was induced with 0.5% compound tropicamide eye drops until adequate dilation was achieved, and topical anesthesia was provided with 0.5% proparacaine hydrochloride. All surgeries were performed by the same experienced surgeon using a standard phacoemulsification technique. A 3.0 mm clear corneal incision was made at a site determined by preoperative corneal topography. Continuous curvilinear capsulorhexis was then performed, followed by phacoemulsification of the lens nucleus and implantation of a Rayner RAO600C intraocular lens into the capsular bag using a preloaded injector system. After surgery, all patients received topical gatifloxacin, prednisolone acetate, and recombinant bovine basic fibroblast growth factor. The dosing schedule was tapered according to the postoperative clinical course.

### 2.4 Definition of primary, secondary and exploratory outcomes

MTF average height was selected as the optical-quality outcome for adjusted analyses because it provides a summary measure of modulation transfer across spatial frequencies and reflects overall optical performance.

The primary outcome was internal MTF average height, which mainly represents the intraocular optical component after lens extraction and IOL implantation.

Total-eye MTF average height and corneal MTF average height were treated as secondary outcomes, reflecting global ocular optical quality and anterior corneal optical component, respectively.

Exploratory analyses included Strehl ratio (SR), higher-order aberrations (HOAs), dysfunctional lens index (DLI), and objective scatter index (OSI). Intergroup differences were compared at each postoperative time point.

### 2.5 Definition of SII

SII is a composite hematologic index that reflects the balance between inflammatory activation and immune status. It is defined as neutrophil count × platelet count / lymphocyte count (all parameters measured in × 10*/L) (19).

### 2.6 Sample size calculation

In this study, linear mixed-effects models were used for data analysis. Two criteria had to be met simultaneously: (1) the total sample size must exceed 100; (2) the sample size should be 10–20 times the number of independent variables. This study primarily investigated the relationship between log2-SII and MTF average height, with adjustment for age, sex, BMI, diabetes, hypertension, and coronary artery disease. A total of seven variables were included. Therefore, the minimum required sample size was 100 (20).

### 2.7 Statistical analysis

Statistical analyses were performed using R software (version 4.3.1). Continuous variables were presented as mean ± standard deviation (SD) or median (p25, p75). Categorical variables are expressed as frequencies and percentages. Due to SII showed a skewed distribution, log2 transformation was applied for analyses. To improve interpretability of the interaction terms, log2-SII was mean-centered. Linear mixed-effects models with a random intercept per patient were used, log2 SII as a continuous variable, postoperative time (1 day, 1 week, 1 month) as a categorical fixed effect, and including the log2-SII * time interaction. Because SII was derived from hematological components and including white blood cells (WBC) might introduce collinearity or overadjustment, WBC was not adjusted for in the primary model. Models were adjusted for age, sex, BMI, diabetes, hypertension and coronary artery disease. Spearman’s correlation was used to evaluate the cross-sectional association between log2-SII and MTF average height at 1 month. In addition, linear regression models were used as Supplementary analyses.

For descriptive purposes only (not for primary inference), patients were divided into low- and high-SII groups based on the median SII. This median-based grouping was used for exploratory between-group comparisons and allowed balanced group sizes. The *t*-test or Mann–Whitney U test were applied for continuous variables. Categorical variables were compared using the χ^2^ test or Fisher’s exact test. Repeated measures ANOVA was used to analyze the effects of SII level and postoperative time on postoperative optical quality.

All tests were two-sided, and *P* < 0.05 was considered statistically significant.

## 3 Results

### 3.1 Baseline characteristics

A total of 110 eyes from 110 patients were finally included in the study. The mean age of the overall cohort was 65.79 ± 10.48 years, the mean BMI was 24.41 ± 2.44 kg/m^2^, 55 (50%) were male and 55 (50%) were female. Patients were evenly divided into low- and high-SII groups according to the median SII value (55 eyes per group). There were no statistically significant differences between the low-SII and high-SII groups regarding age, sex, BMI, marital status, diabetes, hypertension, coronary artery disease, preoperative BCVA, axial length, corneal astigmatism, or corneal endothelial cell density (all *P* > 0.05). Compared to the low-SII group, the high-SII group showed significantly higher white blood cells, SII and log2-SII (all *P* < 0.05). Details were shown in Table 1.

**TABLE 1:** Baseline characteristics of overall patients.

Variable	Overall (*n* = 110)	Low SII group (*n* = 55)	High SII group (*n* = 55)	*P*
Sex, (n%)				0.703
Male	55 (50.00%)	26 (47.27%)	29 (52.73%)	
Female	55 (50.00%)	29 (52.73%)	26 (47.27%)	
Age (years, mean ± SD)	65.79 ± 10.48	67.44 ± 8.19	64.15 ± 12.21	0.100
BMI (kg/m^2^, mean ± SD)	24.41 ± 2.44	24.13 ± 2.66	24.69 ± 2.19	0.234
Marital status, n (%)				0.153
Unmarried/divorced/widowed	14 (12.73%)	4 (7.27%)	10 (18.18%)	
Married	96 (87.27%)	51 (92.73%)	45 (81.82%)	
Diabetes, n (%)				0.639
No	87 (79.09%)	42 (76.36%)	45 (81.82%)	
Yes	23 (20.91%)	13 (23.64%)	10 (18.18%)	
Hypertension, n (%)				0.413
No	75 (68.18%)	40 (72.73%)	35 (63.64%)	
Yes	35 (31.82%)	15 (27.27%)	20 (36.36%)	
Coronary artery disease, n (%)				0.360
No	105 (95.45%)	51 (92.73%)	54 (98.18%)	
Yes	5 (4.55%)	4 (7.27%)	1 (1.82%)	
Operated eye, n (%)				0.849
Left	55 (50.00%)	28 (50.91%)	27 (49.09%)	
Right	55 (50.00%)	27 (49.09%)	28 (50.91%)	
Preoperative BCVA (logMAR, median [IQR])	0.50 (0.3, 0.7)	0.40 (0.30, 0.70)	0.50 (0.30, 0.70)	0.412
Axial length (mm, mean ± SD)	23.73 ± 1.10	23.69 ± 0.98	23.77 ± 1.23	0.685
Corneal astigmatism (D, median [IQR])	0.50 (0.25, 1.00)	0.50 (0.25, 1.00)	0.50 (0.38, 1.00)	0.842
Corneal endothelial cell density (cells/mm^2^, mean ± SD)	2681.77 ± 249.37	2673.09 ± 215.65	2690.45 ± 280.82	0.717
IOL power implanted (D, mean ± SD)	19.65 ± 2.41	19.39 ± 2.66	19.91 ± 2.11	0.259
White blood cells ( × 10^9^/L, mean ± SD)	6.26 ± 1.76	5.13 ± 1.22	7.39 ± 1.46	< 0.001
Red blood cells ( × 10^12^/L, mean ± SD)	4.57 ± 0.48	4.49 ± 0.53	4.66 ± 0.42	0.055
Hemoglobin (g/L, mean ± SD)	141.42 ± 14.34	140.56 ± 14.83	142.27 ± 13.93	0.535
Neutrophils ( × 10^9^/L, median [IQR])	3.67 (2.75, 4.95)	2.74 (2.19, 3.34)	4.93 (4.08, 5.43)	< 0.001
Platelets ( × 10^9^/L, median [IQR])	217.5 (183.5, 257.5)	199.0 (169.5, 225.0)	242.0 (210.5, 273.0)	< 0.001
Lymphocytes ( × 10^9^/L, mean ± SD)	1.81 ± 0.53	1.84 ± 0.52	1.79 ± 0.54	0.637
SII (mean ± SD)	526.51 ± 390.12	300.59 ± 96.68	752.44 ± 440.33	< 0.001
log2-SII (mean ± SD)	8.77 ± 0.88	8.13 ± 0.62	9.42 ± 0.56	< 0.001

Continuous variables were expressed as Mean ± SD or median (IQR) and categorical variables were expressed as NO. (%). BMI, body mass index; BCVA, Best corrected visual acuity; logMAR, Log of the minimum angle of resolution; IOL, Intraocular lens; D, Diopters; SII, systemic immune-inflammation index.

### 3.2 Visual acuity

BCVA improved after surgery in both groups, and no statistically significant between-group differences were observed at any postoperative time point (all P > 0.05). Details are shown in Table 2.

**TABLE 2:** Comparison of BCVA between the low- and high-SII groups at different time points.

Groups	Preoperative	Postoperative		
		1 day	1 week	1 month
Low SII group (*n* = 55)	0.40 (0.30, 0.70)	0.00 (0.00, 0.10)	0.00 (0.00, 0.00)	0.00 (0.00, 0.00)
High SII group (*n* = 55)	0.50 (0.30, 0.70)	0.00 (0.00, 0.10)	0.00 (0.00, 0.00)	0.00 (0.00, 0.00)
*P*	0.412	0.294	1.000	0.897

### 3.3 MTF average height in the early postoperative period

**FIGURE 2:**
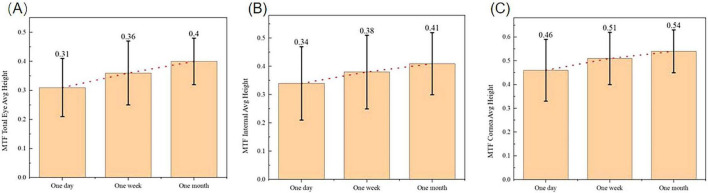
MTF average height in the early postoperative period. **(A)** Total-eye. **(B)** Internal. **(C)** Corneal.

As shown in Figure 2, the MTF average height showed a steady upward trend from 1 day to 1 month postoperatively across all optical components, reflecting a gradual improvement in objective optical performance during the early postoperative period. Total-eye MTF (Figure 2A) increased from 0.31 ± 0.10 at 1 day to 0.40 ± 0.08 at 1 month. Internal MTF (Figure 2B) increased from 0.34 ± 0.13 at 1 day to 0.41 ± 0.11 at 1 month. Corneal MTF (Figure 2C) also increased from 0.46 ± 0.13 at 1 day to 0.54 ± 0.09 at 1 month.

### 3.4 Relationship between SII and MTF average height

For the primary outcome (internal MTF average height), Table 3 presented the associations between log2-SII and postoperative internal MTF average height. Internal MTF average height improved significantly from 1 day to 1 week (β = 0.045, *P* < 0.001) and to 1 month (β = 0.077, *P* < 0.001). No significant main effect of log2-SII was detected at day 1 (β = −0.014, *P* = 0.299). The log2-SII * time interactions were significant at 1 week (β = −0.028, *P* = 0.032) and borderline at 1 month (β = −0.025, *P* = 0.043). Simple slopes revealed that higher log2-SII was associated with lower internal MTF at 1 week (β = −0.042, 95% CI: −0.069 to −0.015, *P* = 0.002) and 1 month (β = −0.039, 95% CI: −0.066 to −0.013, *P* = 0.004), but not at 1 day (*P* = 0.299).

**TABLE 3:** Linear mixed-effects model results for MTF average height.

	Fixed effect	Estimate (95%CI)	*P*
The primary outcome			
Internal	Main effects	–0.014 (–0.041, 0.013)	0.299
	log2-SII (at 1 day)		
	Time (1 week vs. 1 day)	0.045 (0.023, 0.067)	< 0.001
	Time (1 month vs. 1 day)	0.077 (0.055, 0.099)	< 0.001
	Interactions	–0.028 (–0.053, –0.003)	0.032
	log2-SII * 1 week		
	log2-SII * 1 month	–0.025 (–0.050, 0.000)	0.043
	Simple slopes (effect of log2-SII at each time point)		
	1 day	–0.014 (–0.041, 0.013)	0.299
	1 week	–0.042 (–0.069, –0.015)	0.002
	1 month	–0.039 (–0.066, –0.013)	0.004
The secondary outcome			
Total-eye
	Main effects	–0.010 (–0.032, 0.011)	0.351
	log2-SII (at 1 day)		
	Time (1 week vs. 1 day)	0.042 (0.023, 0.062)	< 0.001
	Time (1 month vs. 1 day)	0.084 (0.065, 0.103)	< 0.001
	Interactions	–0.017 (–0.039, 0.006)	0.145
	log2-SII * 1 week		
	log2-SII * 1 month	–0.011 (–0.033, 0.011)	0.336
	Simple slopes (effect of log2-SII at each time point)		
	1 day	–0.010 (–0.032, 0.012)	0.350
	1 week	–0.027 (–0.049, –0.005)	0.016
	1 month	–0.021 (–0.043, 0.001)	0.056
Corneal		
			Main effects
	log2-SII (at 1 day)	–0.008 (–0.032, 0.015)	0.486
	Time (1 week vs. 1 day)	0.043 (0.016, 0.070)	0.002
	Time (1 month vs. 1 day)	0.077 (0.050, 0.104)	< 0.001
	Interactions	0.002 (–0.029, 0.033)	0.880
	log2-SII * 1 week		
	log2-SII * 1 month	–0.020 (–0.051, 0.011)	0.216
	Simple slopes (effect of log2-SII at each time point)		
	1 day	–0.008 (–0.032, 0.015)	0.486
	1 week	–0.006 (–0.030, 0.018)	0.620
	1 month	–0.028 (–0.052, –0.004)	0.021

This table presented the associations between log2-SII and postoperative optical quality, measured by the MTF average height for the total eye, internal and cornea. To improve interpretability of the interaction terms, log2-SII was mean-centered. A linear mixed-effects model with a random intercept per patient was used, adjusting for age, sex, BMI, diabetes, hypertension and coronary artery disease. Postoperative time (1 day, 1 week, 1 month) was treated as a categorical variable, and the log2-SII × time interaction was included. Simple slopes represent the effect of log2-SII at each time point.

For the secondary outcomes (total-eye and corneal MTF average height), the linear mixed-effects models did not show significant interactions between log2-SII and time (total-eye: P for interaction = 0.145 at 1 week and 0.336 at 1 month; corneal: *P* = 0.880 at 1 week and 0.216 at 1 month). Simple slope analysis revealed a negative association at 1 week for total-eye MTF (β = –0.027, 95% CI: –0.049 to –0.005, *P* = 0.016) and at 1 month for corneal MTF (β = –0.028, 95% CI: –0.052 to –0.004, *P* = 0.021).

We also performed exploratory analyses of total-eye (Supplementary Table 1), internal (Supplementary Table 2) and corneal (Supplementary Table 3) MTF average height based on median-split grouping (low-SII vs. high-SII) using repeated-measures ANOVA. The total-eye MTF parameters at different spatial frequencies at various postoperative time points were shown in Supplementary Table 4, internal MTF parameters were shown in Supplementary Table 5 and corneal MTF parameters were shown in Supplementary Table 6.

**FIGURE 3:**
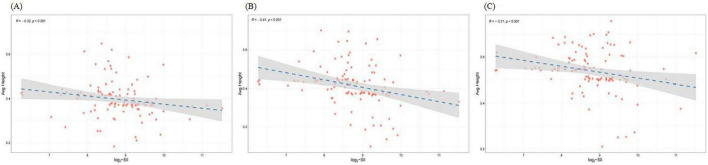
Correlation between log2-SII and MTF average height at 1 month. **(A)** Total-eye. **(B)** Internal. **(C)** Corneal.

In addition, Spearman’s analysis were employed to assess the correlation between SII and MTF at 1 month (Figure 3). log2-SII was negatively correlated with total-eye MTF average height (Figure 3A, *r* = –0.32, *P* < 0.001), internal MTF average height (Figure 3B, *r* = –0.41, *P* < 0.001), and corneal MTF average height (Figure 3C, *r* = –0.31, *P* < 0.001).

Additional cross-sectional linear regression analyses examining the association between log2-SII and internal MTF average height at each postoperative time point were presented in Supplementary Table 7.

### 3.5 Exploratory analysis

#### 3.5.1 Relationship between SII and SR

As shown in Table 4, no significant differences in SR were observed between the low- and high-SII groups at 1 day for any optical component. The internal SR was higher in the low-SII group compared with the high-SII group (0.26 ± 0.16 vs. 0.21 ± 0.11, *P* = 0.036) at 1 week. At 1 month, the low-SII group demonstrated higher SR values than the high-SII group, including total-eye (0.26 ± 0.09 vs. 0.22 ± 0.07, *P* = 0.005), internal (0.30 ± 0.09 vs. 0.25 ± 0.07, *P* = 0.001) and corneal (0.42 ± 0.08 vs. 0.39 ± 0.08, *P* = 0.027).

**TABLE 4:** Comparison of SR between the low- and high-SII groups at different postoperative time points.

Variable	1 day			1 week			1 month		
	Low SII group (*n* = 55)	High SII group (*n* = 55)	*P*	Low SII group (*n* = 55)	High SII group (*n* = 55)	*P*	Low SII group (*n* = 55)	High SII group (*n* = 55)	*P*
Total eye	0.14 ± 0.08	0.13 ± 0.09	0.470	0.21 ± 0.12	0.18 ± 0.09	0.168	0.26 ± 0.09	0.22 ± 0.07	0.005
Internal	0.19 ± 0.15	0.16 ± 0.15	0.309	0.26 ± 0.16	0.21 ± 0.11	0.036	0.30 ± 0.09	0.25 ± 0.07	0.001
Corneal	0.30 ± 0.17	0.30 ± 0.15	0.983	0.37 ± 0.18	0.35 ± 0.11	0.519	0.42 ± 0.08	0.39 ± 0.08	0.027

*P*< 0.05 are considered statistically significant. SII, systemic immune-inflammation index; SR, Strehl ratio.

#### 3.5.2 Relationship between SII and HOAs

At 1 day, no significant between-group differences were observed for any HOA parameter (all *P* > 0.05). At 1 week, only secondary astigmatism differed significantly between groups (*P* = 0.019). At 1 month, the high-SII group exhibited higher total HOA (*P* = 0.003) and secondary astigmatism (*P* = 0.007), while no statistically significant differences were found in coma, spherical aberration and trefoil. Details were shown in Table 5.

**TABLE 5:** Comparison of HOAs between the low- and high-SII groups at different postoperative time points.

Variable	1 day			1 week			1 month		
	Low SII group (*n* = 55)	High SII group (*n* = 55)	*P*	Low SII group (*n* = 55)	High SII group (*n* = 55)	*P*	Low SII group (*n* = 55)	High SII group (*n* = 55)	*P*
HO total	0.29 (0.19, 0.43)	0.34 (0.18, 0.51)	0.626	0.17 (0.14, 0.26)	0.24 (0.16, 0.38)	0.055	0.15 (0.12, 0.20)	0.22 (0.12, 0.36)	0.003
Coma	0.11 (0.06, 0.16)	0.13 (0.07, 0.22)	0.216	0.08 (0.05, 0.13)	0.11 (0.05, 0.17)	0.064	0.07 (0.06, 0.11)	0.08 (0.06, 0.10)	0.931
Spherical	0.03 (0.01, 0.07)	0.04 (0.02, 0.09)	0.092	0.02 (0.01, 0.04)	0.02 (0.01, 0.03)	0.620	0.02 (0.01, 0.04)	0.02 (0.01, 0.03)	0.507
SA	0.04 (0.02, 0.07)	0.06 (0.03, 0.10)	0.087	0.03 (0.01, 0.06)	0.05 (0.02, 0.12)	0.019	0.02 (0.02, 0.03)	0.03 (0.02, 0.04)	0.007
Trefoil	0.19 (0.12, 0.31)	0.21 (0.11, 0.41)	0.685	0.12 (0.08, 0.20)	0.15 (0.09, 0.27)	0.119	0.09 (0.08, 0.14)	0.11 (0.07, 0.14)	0.354

Group comparisons were performed using the Mann–Whitney U test. *P* < 0.05 are considered statistically significant. HOAs, higher-order aberration; SII, systemic immune-inflammation index; SA, secondary astigmatism.

#### 3.5.3 Relationship between SII and DLI, OSI

Table 6 summarized postoperative DLI and OSI by SII group. At 1 day, no differences were observed. At 1 week, the low-SII group demonstrated a significantly higher DLI (7.00 ± 2.01 vs. 5.72 ± 2.40, *P* = 0.003). At 1 month, the low-SII group exhibited higher DLI (7.99 ± 1.39 vs. 7.09 ± 2.03, *P* = 0.008) and lower OSI (1.28 ± 0.34 vs. 1.46 ± 0.48, *P* = 0.030) compared with the high-SII group.

**TABLE 6:** Comparison of DLI and OSI between the low- and high-SII groups at different postoperative time points.

Variable	1 day			1 week			1 month		
	Low SII group	High SII group	*P*	Low SII group	High SII group	*P*	Low SII group	High SII group	*P*
	(*N* = 55)	(*N* = 55)		(*N* = 55)	(*N* = 55)		(*N* = 55)	(*N* = 55)	
DLI	5.44 ± 1.91	4.99 ± 2.30	0.262	7.00 ± 2.01	5.72 ± 2.40	0.003	7.99 ± 1.39	7.09 ± 2.03	0.008
OSI	2.71 ± 1.54	2.99 ± 1.69	0.363	1.70 ± 0.85	1.78 ± 0.78	0.583	1.28 ± 0.34	1.46 ± 0.48	0.030

*P* < 0.05 are considered statistically significant. SII, systemic immune-inflammation index; DLI, dysfunctional lens index; OSI, objective scatter index.

## 4 Discussion

MTF is an important objective metrics commonly used in clinical practice to assess the restoration of visual quality following cataract surgery. Other metrics, such as SR and DLI are also used clinically (21–23). In this study, preoperative SII was associated with differences in early postoperative optical recovery after uncomplicated phacoemulsification. The primary finding was that higher log2-SII was negatively associated with internal MTF average height at postoperative week 1 and month 1 after covariate adjustment. Exploratory analyses suggested that patients with higher SII tended to show lower SR and DLI, as well as higher HOAs and OSI.

This interpretation is clinically plausible. Although modern phacoemulsification is well established and minimally invasive, it still induces transient postoperative inflammation (24). During surgery, ultrasonic energy, corneal incision, and mechanical manipulation of anterior uveal tissues can disrupt the blood–aqueous barrier. This disruption may increase aqueous protein leakage and trigger postoperative inflammatory responses (24, 25). Clinical evidence suggests that, even after uncomplicated cataract surgery, anterior chamber inflammation may not resolve immediately (26). Objective indicators, such as aqueous flare and intraocular inflammatory mediators, can remain elevated for months or even years (27). Such inflammation is not merely a laboratory observation. Increased postoperative aqueous flare has been linked to subsequent macular thickening and pseudophakic cystoid macular edema in clinical studies (28, 29). Even in the absence of overt complications, surgery may still induce subtle and sustained changes in the postoperative intraocular microenvironment (30). Against this background, interindividual variation in postoperative optical recovery is not unexpected. In the present study, such variation in the internal MTF was captured by objective optical quality metrics rather than by BCVA, which may explain why both groups achieved similar acuity despite differences in MTF and in exploratory metrics such as SR, HOAs, DLI, and OSI (31). However, because postoperative inflammatory status was not directly measured, these observations should be interpreted as a plausible biological framework rather than evidence that SII directly reflects local intraocular inflammation.

One possible explanation involves the ocular surface. Early optical quality after cataract surgery depends not only on the intraocular optical system but also on tear film stability and corneal surface regularity. Even mild ocular surface disturbance can reduce optical quality and increase aberrational instability (32–34). Cataract surgery–related factors, including corneal incision, intraoperative ocular surface exposure, perioperative antiseptics, and postoperative topical medications, may collectively impose mechanical, chemical, and phototoxic stress on the ocular surface (5). Through their combined effects on corneal epithelial integrity, goblet cell function, and subbasal nerve architecture, these perioperative influences may predispose a subset of patients to postoperative tear film instability and ocular surface inflammation (35). In parallel, systemic inflammatory status appears to correlate with ocular surface involvement: in primary Sjögren’s syndrome, SII has been reported to associate with disease activity and dry eye involvement, supporting the concept that systemic inflammation and local ocular surface immune responses may interact (36). Although dedicated ocular surface parameters were not collected, this pathway may help explain the observed differences in corneal and total-eye optical quality.

In our study, internal SR also showed differences in exploratory analyses. A possible explanation is that postoperative inflammatory activity may influence the stability of the capsular bag-IOL complex. Persistent inflammation can promote fibrotic remodeling of residual lens epithelial cells and subsequent capsular contraction (37). Clinically, capsular contraction syndrome is characterized by capsular opacification and folding and may lead to a range of intraocular lens–related complications, including tilt, decentration, rotation, or displacement, thereby affecting retinal image formation and postoperative visual quality (38). Even subtle IOL tilt, decentration, or rotational instability has been reported to impair MTF, SR, and HOAs despite preserved visual acuity (39). Oxidative stress may further interact with these inflammatory and reparative processes during early recovery. Reactive oxygen species (ROS) can amplify inflammatory responses, whereas inflammation may further increase oxidative stress (40, 41). Surgical trauma and phacoemulsification energy may add to this oxidative burden in the anterior segment (42, 43). Excess ROS may impair corneal epithelial barrier function, promote lipid peroxidation, and destabilize the tear film, thereby disturbing ocular surface homeostasis (44, 45). Because IOL alignment, capsular contraction, and oxidative stress markers were not evaluated in this study, these explanations should be regarded as hypothesis-generating. Nevertheless, they provide complementary local pathways through which systemic background differences reflected by SII might be associated with slower postoperative optical stabilization.

From the perspective of existing literature, most ophthalmic studies involving SII have focused on disease occurrence, inflammatory severity, or overt postoperative complications. Objective optical quality during the early recovery phase after cataract surgery has received much less attention. The present findings extend prior work by showing that a simple preoperative hematologic index may be associated with how quickly optical quality stabilizes after surgery. Importantly, this should not be interpreted as evidence that patients with high SII will have poorer ultimate surgical outcomes. A more cautious interpretation is that they may follow a different early recovery trajectory. Clinically, SII should not be regarded as a standalone prognostic marker, screening tool, or decision-making indicator based on the present data. Rather, it may serve as one background factor for interpreting early postoperative variability and should be considered together with ocular surface status, cataract characteristics, baseline visual function, systemic comorbidities, and perioperative factors. The exploratory findings regarding SR, HOAs, DLI, and OSI should be considered hypothesis-generating and require validation in future studies.

Several limitations should be acknowledged. First, because of the retrospective design, the present study can only describe associations rather than causation. Second, SII is a nonspecific composite index and may be influenced by physiological stress, metabolic status, and other systemic factors not fully captured in the available data. Although patients with documented recent infection, autoimmune disease, hematologic disorders, or corticosteroid/immunomodulatory therapy were excluded, subclinical conditions such as metabolic syndrome, poor glycemic control, thyroid dysfunction, chronic low-grade infection, or undiagnosed Sjögren’s syndrome were not systematically assessed. These factors may affect both SII and ocular surface or corneal recovery, leading to residual confounding. Third, we did not assess postoperative inflammatory status, local inflammatory biomarkers, tear film parameters, subjective visual quality, or direct indicators of IOL position and capsular contraction. Therefore, the proposed mechanisms should be interpreted as hypothesis-generating rather than directly verified pathways. Finally, the follow-up period was restricted to the first postoperative month, and whether these early differences persist or disappear over longer follow-up remains unclear. Future prospective studies with longitudinal assessment of inflammatory status, ocular surface condition, subjective visual quality, and IOL position or capsular bag stability are needed to clarify these relationships.

## 5 Conclusion

This study showed that higher preoperative SII was associated with poorer early recovery of objective optical quality after cataract surgery. This association was particularly evident for internal MTF average height. Exploratory analyses of SR, HOAs, DLI, and OSI showed similar trends, but these findings should be interpreted cautiously. Overall, the results suggest that elevated preoperative SII may reflect a systemic background related to a different early recovery trajectory.

## Data Availability

The original contributions presented in this study are included in the article/Supplementary material, further inquiries can be directed to the corresponding author.
