# Supplementary material for: Association between systemic immune-inflammation index and postoperative optical quality in the early recovery phase after cataract surgery: a retrospective study
Source: Front Med (Lausanne). 2026 Jun 12;13:1837927. doi: 10.3389/fmed.2026.1837927 (PMC13325468; doi:10.3389/fmed.2026.1837927)
Supplement: Supplementary file 1 [file Table_1.docx]

**Table S1 Comparison of total-eye MTF average height at different postoperative time points between different SII groups**

|  |  |  |  | Time | | Group | | Time*Group | |
| --- | --- | --- | --- | --- | --- | --- | --- | --- | --- |
| Group | One day | One week | One month | F | P | F | P | F | P |
| Low SII | 0.32 ± 0.09 | 0.38 ± 0.11 | 0.42 ± 0.09 | 36.729 | <0.001 | 4.369 | 0.039 | 1.767 | 0.173 |
| High SII | 0.31 ± 0.12 | 0.33 ± 0.11 | 0.38 ± 0.07 |  |  |  |  |  |  |
| P | 0.561 | 0.023 | 0.025 |  |  |  |  |  |  |

**Table S2 Comparison of internal MTF average height at different postoperative time points between different SII groups**

|  |  |  |  | Time | | Group | | Time*Group | |
| --- | --- | --- | --- | --- | --- | --- | --- | --- | --- |
| Group | One day | One week | One month | F | P | F | P | F | P |
| Low SII | 0.36 ± 0.13 | 0.42 ± 0.12 | 0.45 ± 0.10 | 23.323 | <0.001 | 10.460 | 0.002 | 1.838 | 0.162 |
| High SII | 0.32 ± 0.14 | 0.35 ± 0.12 | 0.38 ± 0.11 |  |  |  |  |  |  |
| P | 0.154 | 0.004 | <0.001 |  |  |  |  |  |  |

**Table S3 Comparison of corneal MTF average height at different postoperative time points between different SII groups**

|  |  |  |  | Time | | Group | | Time*Group | |
| --- | --- | --- | --- | --- | --- | --- | --- | --- | --- |
| Group | One day | One week | One month | F | P | F | P | F | P |
| Low SII | 0.47 ± 0.14 | 0.51 ± 0.11 | 0.56 ± 0.07 | 15.410 | <0.001 | 1.411 | 0.238 | 0.771 | 0.464 |
| High SII | 0.46 ± 0.13 | 0.50 ± 0.10 | 0.52 ± 0.11 |  |  |  |  |  |  |
| P | 0.844 | 0.667 | 0.041 |  |  |  |  |  |  |

**Table S4 Comparison of MTF total eye parameters between the low- and high-SII groups at different postoperative time points**

|  | 1 day | | | 1 week | | | 1 month | | |
| --- | --- | --- | --- | --- | --- | --- | --- | --- | --- |
| Variable | Low SII group (n=55) | High SII group (n=55) | P | Low SII group (n=55) | High SII group (n=55) | P | Low SII group (n=55) | High SII group (n=55) | P |
| 5 cycles/degree | 0.59 ± 0.16 | 0.55 ± 0.22 | 0.404 | 0.67 ± 0.16 | 0.59 ± 0.20 | 0.029 | 0.72 ± 0.10 | 0.67 ± 0.13 | 0.031 |
| 10 cycles/degree | 0.31 ± 0.14 | 0.30 ± 0.17 | 0.660 | 0.41 ± 0.16 | 0.34 ± 0.16 | 0.029 | 0.46 ± 0.12 | 0.41 ± 0.12 | 0.068 |
| 15 cycles/degree | 0.20 ± 0.10 | 0.19 ± 0.12 | 0.775 | 0.27 ± 0.13 | 0.23 ± 0.13 | 0.149 | 0.30 ± 0.11 | 0.26 ± 0.12 | 0.077 |
| 20 cycles/degree | 0.14 ± 0.07 | 0.13 ± 0.07 | 0.336 | 0.20 ± 0.10 | 0.17 ± 0.10 | 0.192 | 0.23 ± 0.09 | 0.21 ± 0.09 | 0.132 |
| 25 cycles/degree | 0.11 ± 0.05 | 0.10 ± 0.05 | 0.134 | 0.16 ± 0.08 | 0.14 ± 0.08 | 0.231 | 0.18 ± 0.07 | 0.16 ± 0.07 | 0.190 |
| 30 cycles/degree | 0.09 ± 0.05 | 0.08 ± 0.05 | 0.280 | 0.13 ± 0.07 | 0.11 ± 0.07 | 0.223 | 0.15 ± 0.06 | 0.13 ± 0.06 | 0.181 |

P values less than 0.05 are considered statistically significant. SII, systemic immune-inflammation index; MTF, modulation transfer function.

**Table S5 Comparison of MTF internal parameters between the low- and high-SII groups at different postoperative time points**

|  | 1 day | | | 1 week | | | 1 month | | |
| --- | --- | --- | --- | --- | --- | --- | --- | --- | --- |
| Variable | Low SII group (n=55) | High SII group (n=55) | P | Low SII group (n=55) | High SII group (n=55) | P | Low SII group (n=55) | High SII group (n=55) | P |
| 10 cycles/degree | 0.37 ± 0.18 | 0.32 ± 0.20 | 0.166 | 0.46 ± 0.17 | 0.37 ± 0.18 | 0.008 | 0.49 ± 0.16 | 0.41 ± 0.17 | 0.014 |
| 15 cycles/degree | 0.24 ± 0.15 | 0.21 ± 0.15 | 0.261 | 0.31 ± 0.15 | 0.24 ± 0.13 | 0.006 | 0.34 ± 0.14 | 0.27 ± 0.13 | 0.007 |
| 20 cycles/degree | 0.18 ± 0.12 | 0.16 ± 0.12 | 0.449 | 0.23 ± 0.13 | 0.19 ± 0.17 | 0.118 | 0.26 ± 0.12 | 0.20 ± 0.10 | 0.005 |
| 25 cycles/degree | 0.14 ± 0.10 | 0.13 ± 0.11 | 0.604 | 0.18 ± 0.11 | 0.15 ± 0.07 | 0.036 | 0.20 ± 0.10 | 0.16 ± 0.08 | 0.007 |
| 30 cycles/degree | 0.12 ± 0.08 | 0.10 ± 0.08 | 0.214 | 0.16 ± 0.09 | 0.11 ± 0.07 | 0.005 | 0.17 ± 0.09 | 0.13 ± 0.07 | 0.008 |

P values less than 0.05 are considered statistically significant. SII, systemic immune-inflammation index; MTF, modulation transfer function.

**Table S6 Comparison of MTF corneal parameters between the low- and high-SII groups at different postoperative time points**

|  | 1 day | | | 1 week | | | 1 month | | |
| --- | --- | --- | --- | --- | --- | --- | --- | --- | --- |
| Variable | Low SII group (n=55) | High SII group (n=55) | P | Low SII group (n=55) | High SII group (n=55) | P | Low SII group (n=55) | High SII group (n=55) | P |
| 5 cycles/degree | 0.75 ± 0.14 | 0.71 ± 0.15 | 0.162 | 0.80 ± 0.10 | 0.78 ± 0.15 | 0.402 | 0.83 ± 0.06 | 0.79 ± 0.11 | 0.030 |
| 10 cycles/degree | 0.51 ± 0.19 | 0.50 ± 0.16 | 0.781 | 0.58 ± 0.15 | 0.57 ± 0.14 | 0.678 | 0.63 ± 0.11 | 0.60 ± 0.15 | 0.181 |
| 15 cycles/degree | 0.38 ± 0.18 | 0.37 ± 0.14 | 0.906 | 0.44 ± 0.15 | 0.42 ± 0.12 | 0.585 | 0.47 ± 0.12 | 0.45 ± 0.14 | 0.470 |
| 20 cycles/degree | 0.28 ± 0.13 | 0.27 ± 0.10 | 0.558 | 0.34 ± 0.14 | 0.33 ± 0.10 | 0.761 | 0.39 ± 0.09 | 0.37 ± 0.13 | 0.353 |
| 25 cycles/degree | 0.21 ± 0.10 | 0.19 ± 0.07 | 0.191 | 0.27 ± 0.12 | 0.26 ± 0.08 | 0.433 | 0.31 ± 0.09 | 0.29 ± 0.11 | 0.205 |
| 30 cycles/degree | 0.18 ± 0.08 | 0.16 ± 0.06 | 0.165 | 0.23 ± 0.11 | 0.21 ± 0.07 | 0.186 | 0.26 ± 0.07 | 0.25 ± 0.10 | 0.591 |

P values less than 0.05 are considered statistically significant. SII, systemic immune-inflammation index; MTF, modulation transfer function.

**Table S7 Relationship between log2-SII and internal MTF average height (Cross-sectional analysis)**

In the overall patients, univariate logistic regression results (Model 1) showed log2-SII was significantly negatively correlated with the internal MTF average height at one week (β: -0.04, 95% CI: -0.07, -0.01, P=0.003) and one month (β: -0.04, 95% CI: -0.06, -0.01, P=0.002) postoperatively. After adjusting for age, sex and BMI inModel 2, this relationship remained (one week: β= -0.04, 95% CI: -0.07, -0.02, P=0.002; one month: β= -0.04, 95% CI: -0.06, -0.01, P=0.002). After adjusting for all covariates in Model 3, log2-SII remained significantly negatively associated with the internal MTF average height (one week: β= -0.05, 95% CI: -0.07, -0.02, P=0.002; one month: β= -0.04, 95% CI: -0.06, -0.01, P=0.002). In addition, although there was no statistically significant difference between log2-SII and the internal MTF average height at one day postoperatively, the results suggested a negative correlation between log2-SII and the internal MTF average height.

| Postoperative time points | Model 1 | | Model 2 | | Model 3 | |
| --- | --- | --- | --- | --- | --- | --- |
|  | β (95%CI) | P | β (95%CI) | P | β (95%CI) | P |
| 1 day | -0.01 (-0.04 ~ 0.02) | 0.407 | -0.02 (-0.04 ~ 0.01) | 0.286 | -0.01 (-0.04 ~ 0.02) | 0.374 |
| 1 week | -0.04 (-0.07 ~ -0.01) | 0.003 | -0.04 (-0.07 ~ -0.02) | 0.002 | -0.05 (-0.07 ~ -0.02) | 0.002 |
| 1 month | -0.04 (-0.06 ~ -0.01) | 0.002 | -0.04 (-0.06 ~ -0.01) | 0.002 | -0.04 (-0.06 ~ -0.01) | 0.003 |

95% CI, 95% conﬁdence interval.

Model 1: no covariates were adjusted.

Model 2: adjusted for age, sex and BMI.

Model 3: adjusted for age, sex, BMI, marital status, diabetes, hypertension, coronary artery disease, white blood cells, red blood cells and hemoglobin.
